# ADCK1 activates the β-catenin/TCF signaling pathway to promote the growth and migration of colon cancer cells

**DOI:** 10.1038/s41419-021-03624-9

**Published:** 2021-04-06

**Authors:** Yong Ji, Yiqian Liu, Changchun Sun, Lijiang Yu, Zhao Wang, Xu Du, Wu Yang, Chenggong Zhang, Chunmu Tao, Jianjiang Wang, Xi Yang, Sun Di, Yufeng Huang

**Affiliations:** 1 Department of General Surgery, Jingjiang People’s Hospital Affiliated with Yangzhou University, Jingjiang, China; 2 Department of Oncology, First Affiliated Hospital of Nanjing Medical University, 210029 Nanjing, China; 3 Department of Oncology, Jingjiang People’s Hospital Affiliated with Yangzhou University, 214500 Jingjiang, China; 4 Department of Pathology, Jingjiang People’s Hospital Affiliated with Yangzhou University, 214500 Jingjiang, China; 5 Department of Radiation Oncology, Fudan University Shanghai Cancer Center, 200032 Shanghai, China

**Keywords:** Cancer models, Gastrointestinal cancer

## Abstract

As a result of mutations in the upstream components of the Wnt/β-catenin signaling pathway, this cascade is abnormally activated in colon cancer. Hence, identifying the activation mechanism of this pathway is an urgent need for the treatment of colon cancer. Here, we found an increase in ADCK1 (AarF domain-containing kinase 1) expression in clinical specimens of colon cancer and animal models. Upregulation of ADCK1 expression promoted the colony formation and infiltration of cancer cells. Downregulation of ADCK1 expression inhibited the colony formation and infiltration of cancer cells, in vivo tumorigenesis, migration, and organoid formation. Molecular mechanistic studies demonstrated that ADCK1 interacted with TCF4 (T-cell factor 4) to activate the β-catenin/TCF signaling pathway. In conclusion, our research revealed the functions of ADCK1 in the development of colon cancer and provided potential therapeutic targets.

## Introduction

Colon cancer is one of the most common malignancies^1^. During its formation and progression, colon cancer may result in many changes at the genetic level, such as Kras-activating mutations, P53 inactivating mutations, and abnormal activation of the Wnt/β-catenin signaling pathway^2^. In ~90% of familial and sporadic colon cancers, APC, the upstream component of the Wnt/β-catenin signaling pathway, has a truncation mutation^3^. Additionally, the core transcription factor β-catenin is mutated in colon cancer, and the mutated β-catenin protein is more stable^4^. Therefore, studying the regulation of the β-catenin signaling pathway in colon cancer is of great importance for colon cancer treatment.

The core molecule in this pathway is β-catenin^5^. In resting cells, the protein level of β-catenin is strictly controlled by a degradation complex. The destruction complex is composed of APC, Axin, GSK3β, and CK1α and is responsible for phosphorylating the N-terminus of β-catenin, consequently leading to the degradation of β-catenin. If stimulated by Wnt ligands, the degradation complex dissociates, and β-catenin accumulates in the cytoplasm as a result. The accumulated β-catenin migrates into the cell nucleus and forms a complex with the transcription factor TCF4, which can activate the transcription of some downstream genes (Axin2, c-Myc, Cyclin D1, etc.)^6^. Wnt/β-catenin directly drives the progression of enteric epithelium from polyposis to adenocarcinoma^3^. Therefore, safe and effective intervention with this signaling pathway is of critical importance in the treatment of colon cancer. Considering the mutation of the upstream mediator APC and β-catenin, the transcription complex in the cell nucleus is a perfect intervention target.

There is still limited knowledge of the biological functions of ADCK1 at present. In Drosophila, ADCK1 is critical for maintaining mitochondrial structures and functions in muscle, and knocking out ADCK1 in Drosophila results in severe developmental defects^7,8^. Additionally, genetic polymorphisms in ADCK1 are closely related to schizophrenia and can be used for predicting the curative effects of paliperidone, a therapeutic drug for schizophrenia^9–11^. The functions of ADCK1 in tumor formation and development remain unknown. Only one study discovered mutation of ADCK1 in parathyroid tumors^10^, but the biological functions of ADCK1 are still unclear.

In this study, we examined the expression of ADCK1 in colon cancer, revealed its functions in intestinal cancer, and provided novel insights into the molecular mechanism of ADCK1 in regulating the Wnt/β-catenin signaling pathway.

## Results

### ADCK1 expression was upregulated in colon cancer

**Fig. 1:**
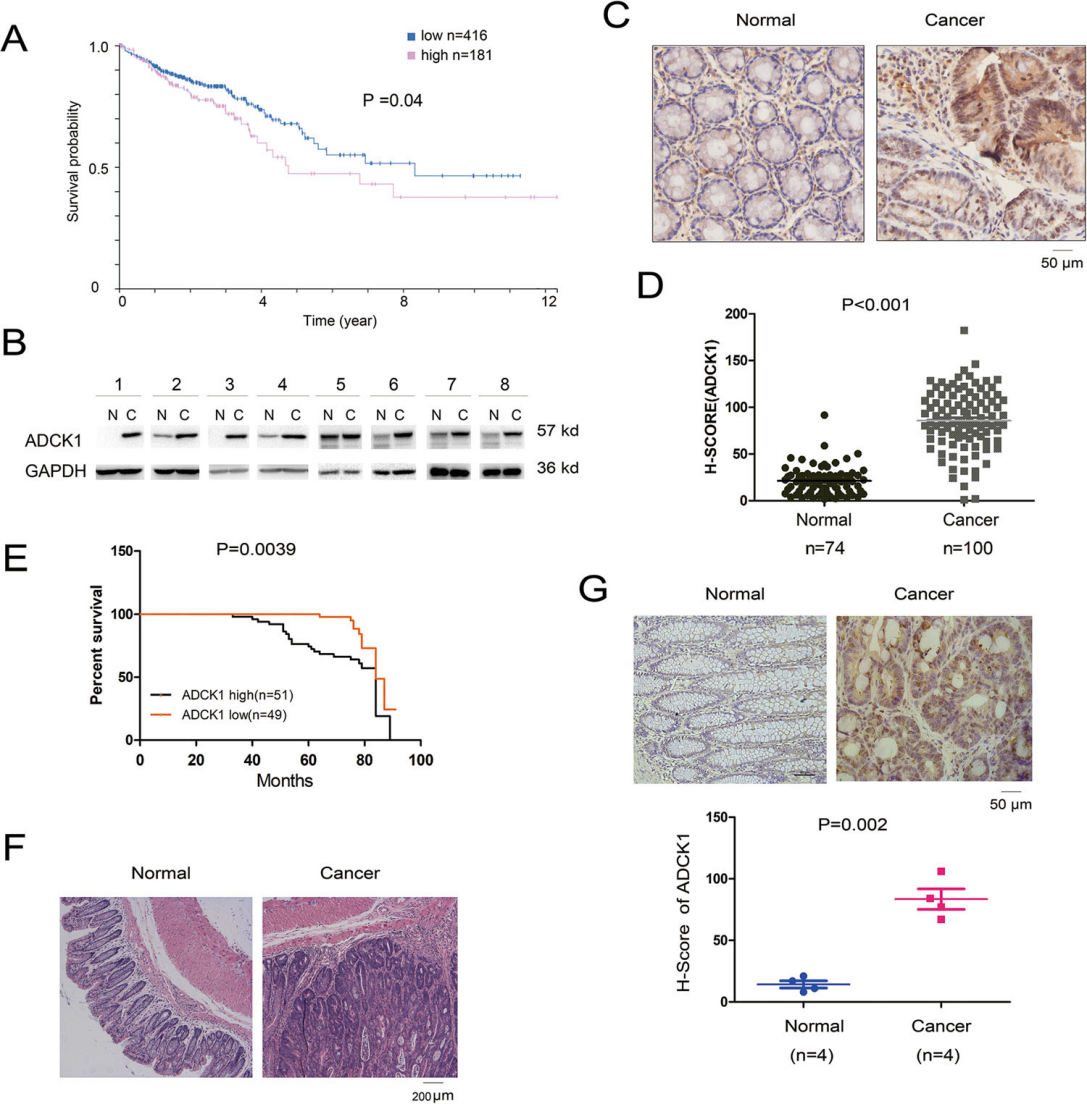
ADCK1 was upregulated in colon cancer. **A** The Human Protein Atlas (HPA) database was used to analyze the correlation between ADCK1 expression and survival. The cohort was divided into two groups, high (*n* = 181) and low (*n* = 416), according to the expression of ADCK1. **B** The protein levels of ADCK1 in colon cancer tissues and paired normal tissues were determined. Tissues were homogenized, and proteins in the tissues were extracted using RIPA buffer. **C**, **D** IHC was performed to examine the protein levels of ADCK1 in the tissue array. Signals were detected using the Vectra 2.0 system, and statistical analysis was performed. **E** Survival analysis was performed. The survival curve was plotted with the Kaplan–Meier method, while the log-rank test was used for analysis. **F** HE staining was performed to examine the tumors in the intestines of APC^min/+^ mice. Left, normal tissues; right, tumor tissues. **G** IHC staining was performed to examine the protein levels of ADCK1 in the tumors shown in **F**. The staining was scored and analyzed. The scale bars were indicated.

To study the expression pattern of ADCK1 in colon cancer, we first searched the HPA (Human Protein Atlas) database and studied the relationship between ADCK1 expression in colon cancer patients and patient survival. The study showed that ADCK1 was negatively correlated with the survival time of the patients (*P* = 0.04) (Fig. 1A). Later, we evaluated ADCK1 expression in eight pairs of cancer tissues and paired paracarcinoma tissues. Seven of the eight pairs of tissues showed high expression of ADCK1 in the tumor tissues (Fig. 1B). We next examined the expression of ADCK1 using a tissue array that contained 77 normal colon epithelial tissues and 100 colon cancer samples. As shown in Fig. 1C, D, upregulation of ADCK1 was observed (*P* < 0.01). Moreover, the expression of ADCK1 was correlated with the tumor stage, metastasis, and the outcome of the chemotherapy regimen (Table 1). Most importantly, survival analysis revealed that high expression of ADCK1 implied a shorter overall survival time (Fig. 1E) (*P* = 0.0039). In addition, multivariate Cox analysis showed that high expression of ADCK1 had a significant prognostic value (Table 2).

**Table 1:** The correlation between the expression of ADCK1 and clinical features.

Characteristic	Total	ADCK1 expression		*χ*^2^	*P*-value
		Low, *n* = 49	High, *n* = 51		
*Gender*					
Male	48	21	27	0.313	0.326
Female	52	28	24		
*Age (years)*					
>60	60	31	29	0.514	0.327
≤60	40	18	22		
*Tumor size (cm*^*3*^*)*					
>50	17	6	11	1.545	0.462
10–50	46	24	22		
<10	37	19	18		
*TNM stage*					
I–II	48	32	16	11.529	0.001
III–IV	52	17	35		
*Organ metastasis*					
Yes	31	10	21	5.039	0.031
No	69	39	30		
*Lymph node metastasis*					
0	11	7	4		
1–5	23	6	17		
≥5	66	36	30	6.587	0.037
*AJCC cancer stage, 7th edition*					
I–II	48	32	16		
III–IV	52	17	35	11.529	0.001
*Reoccurence*					
Yes	26	10	16		
No	74	39	35	0.211	0.257
*Pathologicl grade*					
I, I–II	97	47	50		
II, III	3	2	1		
*Chemotherapy regimen*					
XELOX	53	29	24		
FOLFOX	43	16	27	7.249	0.027

**Table 2:** Multivariate Cox analysis showed that high expression of ADCK1 had significant prognostic value.

	Univariate			Multivariate		
	HR	95% CI	*P*	HR	95% CI	*P*
*ADCK1*			0.007*			0.014*
High	2.882	1.33–6.241		2.679	1.226–5.855	
Low	Ref	Ref		Ref	Ref	
*Distant metastasis*			0.008*			0.016*
Yes	2.612	1.291–5.282		2.373	1.175–4.796	
No	Ref	Ref		Ref	Ref	
*Clinical stages*			0.031*			
III–IV	2.570	1.092–6.049				
I–II	Ref	Ref				
*Lymph node metastasis*			0.658			
Yes	1.173	0.578–2.381				
No	Ref	Ref				
*Chemotherapy regimen*						
XELOX, XELOX + radiation therapy	5740.710	0–7.914E+84	0.928			
FOLFOX, FOLFOX + radiation therapy	14,253.195	0–1.964E+85	0.920			
Others	Ref	Ref				

Variables in equations								
	*B*	Standard error of	Wald	Degrees of freedom	Significant	Exp (*B*)	95% Confidence interval for exp (*B*)	
							The lower limit	Ceiling
*Step 1*								
ADCK1	0.847	0.412	4.228	1	0.040	2.332	1.040	5.226
Metastasis	0.725	0.541	1.792	1	0.046	2.074	1.012	4.252
Clinical stages	0.009	0.749	0.000	1	0.991	1.009	0.233	4.377
Lymph node metastasis	−0.397	0.506	0.614	1	0.433	0.673	0.249	1.814
Chemoradiotherapy regimen			4.384	2	0.112	2.528	1.061	6.024
Chemoradiotherapy regimen (1)	0.928	0.443	4.384	1	0.036	0.000	0.000	
Chemoradiotherapy regimen (2)	−11.189	479.777	0.001	1	0.981	0.000	0.000	–
*Step 2*								
ADCK1	0.848	0.399	4.511	1	0.034	2.335	1.068	5.105
Metastasis	0.730	0.366	3.968	1	0.181	2.064	0.714	5.966
Lymph node metastasis	−0.393	0.412	0.913	1	0.339	0.675	0.301	1.512
Chemoradiotherapy regimen			4.403	2	0.111			
Chemoradiotherapy regimen (1)	0.928	0.442	4.402	1	0.036	2.529	1.063	6.017
Chemoradiotherapy regimen (2)	−11.190	479.754	0.001	1	0.981	0.000	0.000	–
*Step 3*								
ADCK1	0.870	0.397	4.798	1	0.028	2.386	1.096	5.197
Metastasis	0.772	0.363	4.510	1	0.034	2.163	1.061	4.409
Chemoradiotherapy regimen			3.418	2	0.181			
Chemoradiotherapy regimen (1)	0.718	0.389	3.417	1	0.065	2.051	0.958	4.392
Chemoradiotherapy regimen (2)	−11.077	472.043	0.001	1	0.981	0.000	0.000	–
*Step 4*								
ADCK1	0.985	0.399	6.102	1	0.014	2.679	1.226	5.855
Metastasis	0.864	0.359	5.799	1	0.016	2.373	1.175	4.796

*HR* hazard odds, *CI* confidence interval.

*P < 0.05.

The Apc^Min^ mouse is a classical model for studying colon cancer. We first evaluated adenoma formation in this mouse model (Fig. 1F) and examined ADCK1 expression in the adenomas using immunohistochemical staining. Similarly, ADCK1 expression increased in early adenomas (Fig. 1G) (*P* = 0.002).

### ADCK1 promoted the colony formation and invasion of colon cancer cells

**Fig. 2:**
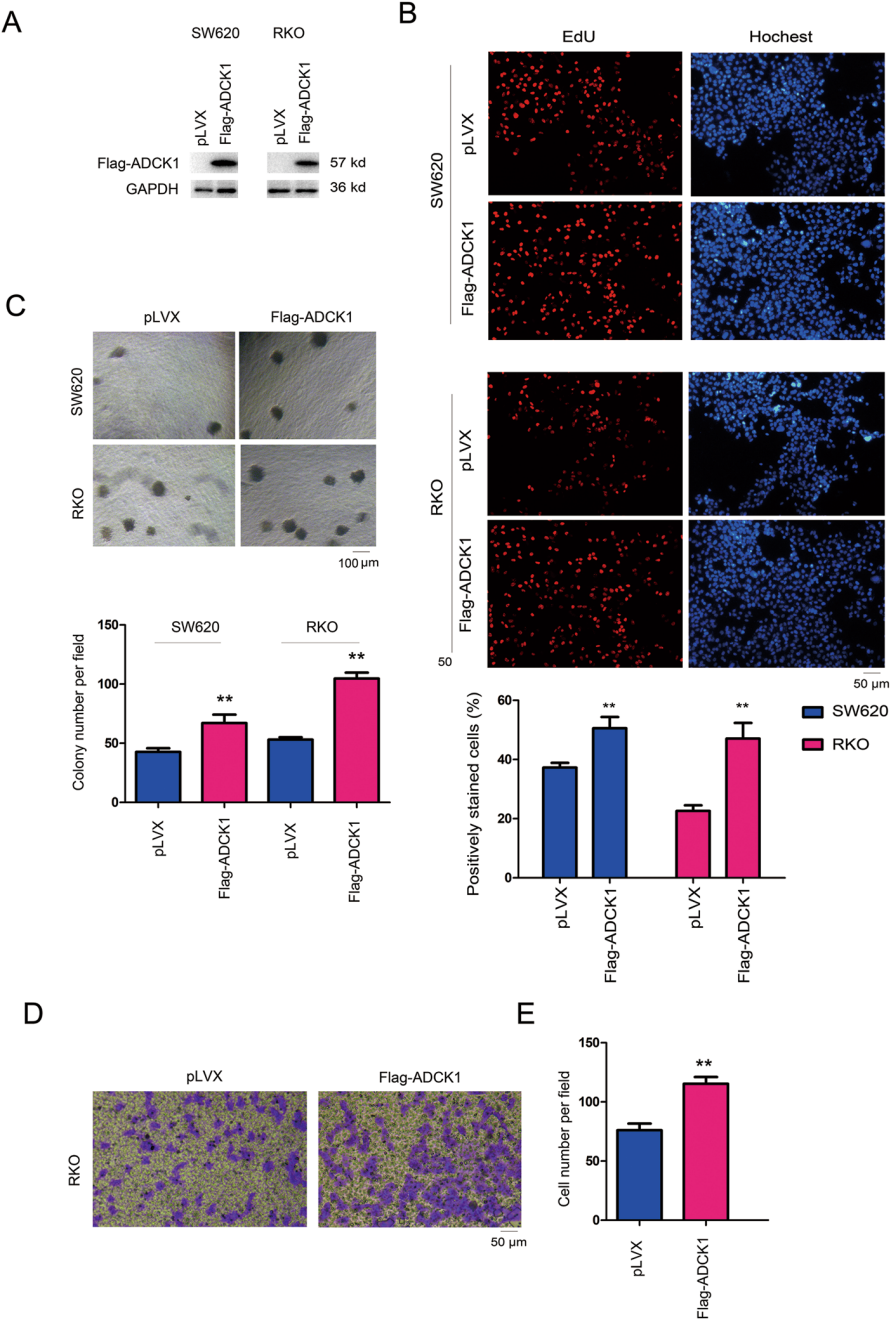
ADCK1 promoted the anchorage-independent growth and invasion of colon cancer cells. **A** Overexpression of ADCK1 in SW620 and RKO cells. Cells were infected with lentivirus, and the expression of exogenous ADCK1 (Flag-ADCK1) was examined using western blotting. **B** An EdU assay was performed to examine the roles of ADCK1 in cell proliferation. The details are described in the “Experimental procedures” section. Percentage of positively stained cells= number of positively stained cells/number of total cells. **C** The effects of ADCK1 overexpression on the anchorage-independent growth of SW620 and RKO cells were evaluated using a soft agar assay. The details are described in the “Experimental procedures” section. The colonies were counted, and statistical analysis was performed. **D**, **E** The effects of ADCK1 overexpression on the invasion of RKO cells were evaluated using a Transwell assay. **P < 0.01. The scale bars were indicated.

The results of the experiment with the clinical specimens indicated that the expression of ADCK1 was upregulated in tumor tissues. Therefore, we later selected two colon cancer cell lines, SW620 and RKO, for forced ADCK1 expression (Fig. 2A). Then, EdU (Fig. 2B), soft agar (Fig. 2C) and Transwell (Fig. 2D, E) assays were performed to measure the impact of ADCK1 expression on the proliferation, colony formation and invasion abilities of colon cancer cells. The experiments showed that the upregulated ADCK1 expression in SW620 and RKO cells promoted their ability to proliferate, form colonies on soft agar and invade.

### Downregulated ADCK1 expression inhibited the colony formation and invasion of colon cancer cells

**Fig. 3:**
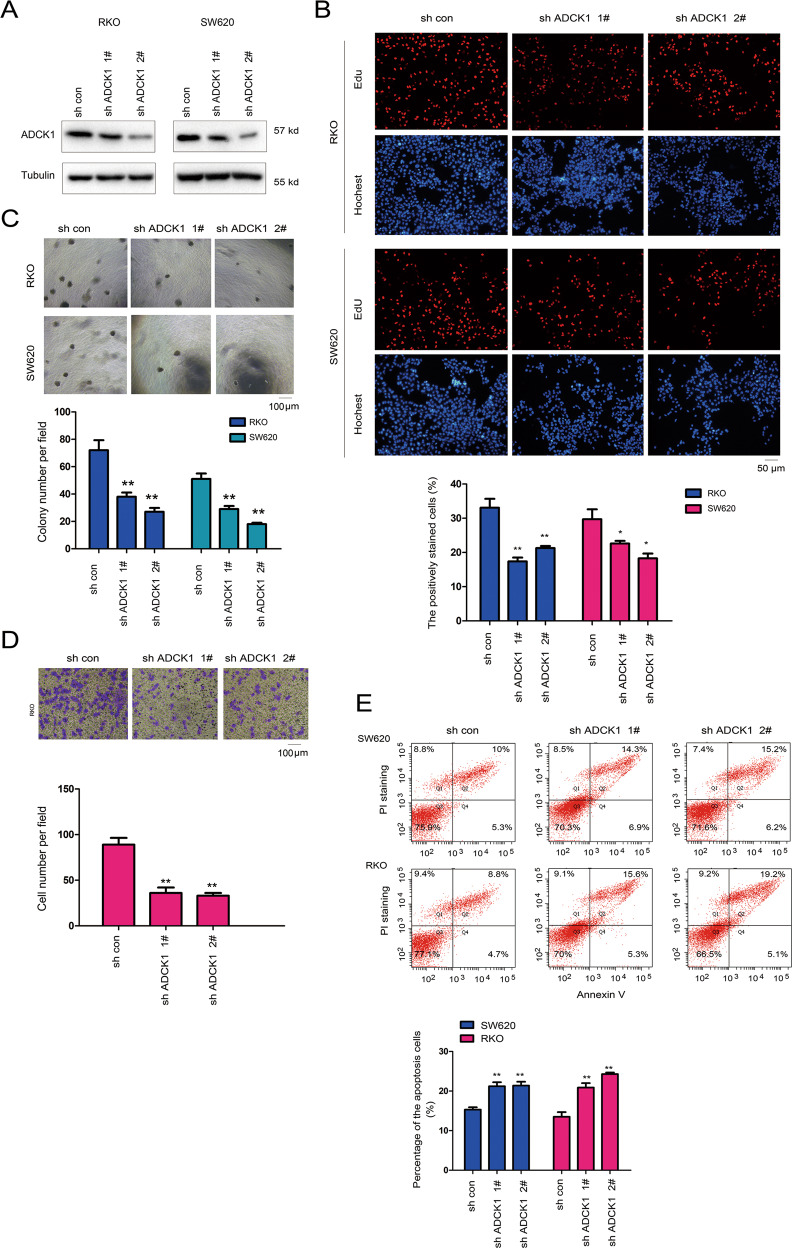
Knockdown of ADCK1 promoted the anchorage-independent growth and invasion of colon cancer cells. **A** Knockdown of ADCK1 in SW620 and RKO cells. The expression of ADCK1 was examined using western blotting. **B** An EdU assay was performed to examine the effects of ADCK1 knockdown on cell proliferation. The details are described in the “Experimental procedures” section. Percentage of positively stained cells = number of positively stained cells/number of total cells. **C** The effects of ADCK1 knockdown on the anchorage-independent growth of SW620 and RKO cells were evaluated using a soft agar assay. **D** The effects of ADCK1 knockdown on the invasion of RKO cells were evaluated using a Transwell assay. The migrated cells were counted, and statistical analysis was performed. **E** The effects of ADCK1 knockdown on RKO cell apoptosis. Cells were fixed with 75% ethanol, stained with PI and Annexin V, and then analyzed by cytometry. The scale bars were indicated. **P < 0.01; *P < 0.05.

Subsequently, we interfered with ADCK1 expression in colon cancer cells (Fig. 3A) and examined the impact of downregulation of ADCK1 expression on the proliferation, colony formation, and invasion of colon cancer cells through EdU, soft agar colony formation, and Transwell assays. The results indicated that downregulation of ADCK1 expression in SW620 and RKO cells compromised their ability to proliferate (Fig. 3B), form colonies (Fig. 3C), and invade (Fig. 3D). Moreover, knockdown of ADCK1 promoted the apoptosis of colon cancer cells (Fig. 3E).

### Knockdown of ADCK1 expression inhibited the formation of organoids and tumor formation

**Fig. 4:**
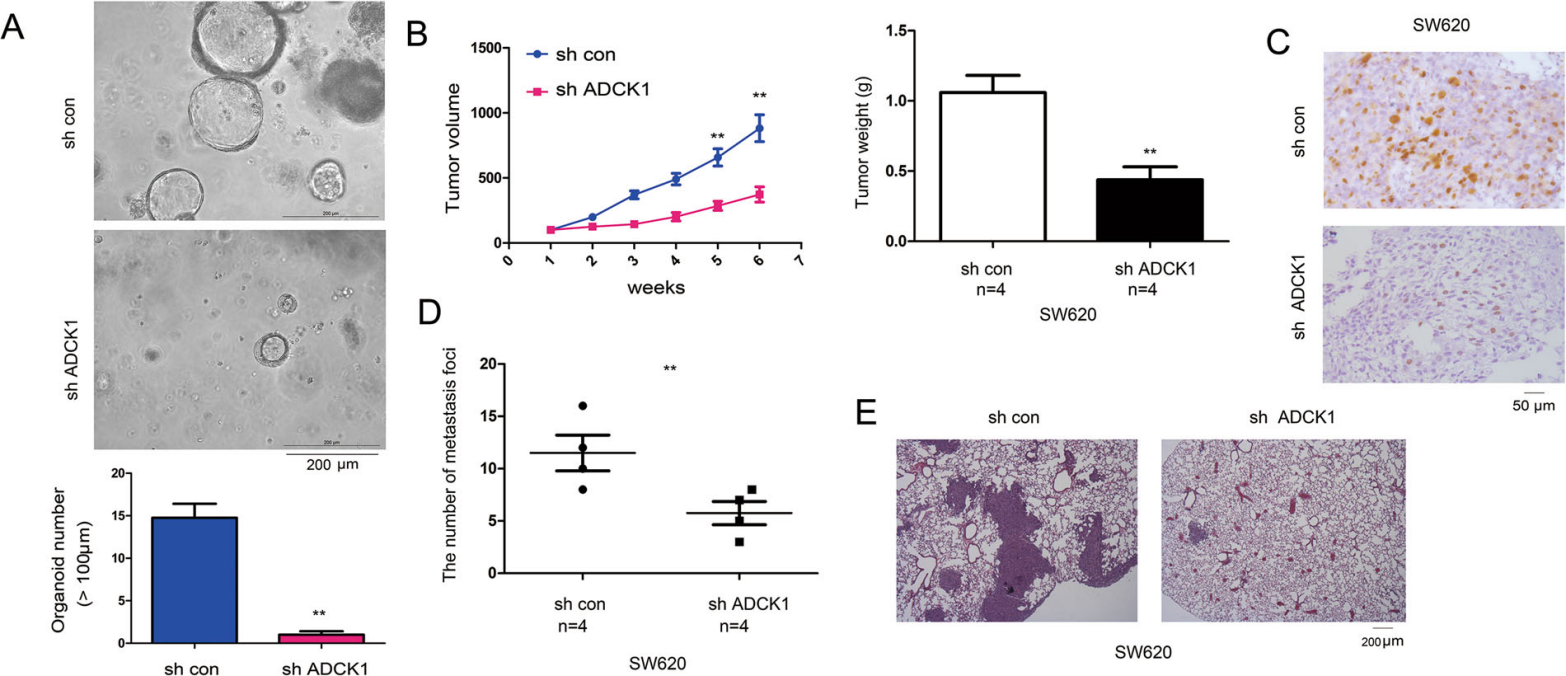
Knockdown of ADCK1 inhibited tumorigenesis. **A** The effects of ADCK1 knockdown on organoid formation were assessed. The tumors of C57BL/6J-*Apc*^Min^/J mice were used for organoid culture as described in the “Experimental procedures” section. The number of organoids (>100 µm) was quantified. **B**, **C** The effects of ADCK1 knockdown on tumor formation by SW620 cells were assessed. SW620 cells with ADCK1 knockdown and control SW620 cells were subcutaneously injected into nude mice. The tumors were harvested, the tumor volume and weight were measured, and IHC was performed to examine the expression of ADCK1. **D**, **E** The effects of ADCK1 knockdown on the distant seeding of SW620 cells were evaluated in nude mice as described in the “Experimental procedures” section. The lungs of the mice were collected, and the metastatic foci were examined using HE staining. The scale bars were indicated. **P < 0.01.

To determine the in vivo functions of ADCK1, we established an organoid model using the tumors tissues harbored by C57BL/6J-ApcMin/J mice. In this model, we knocked down the expression of ADCK1. The experiment showed that the number and the size of the organoids decreased after the knockdown of ADCK1 expression (Fig. 4A). In the subcutaneous tumor formation assay, we found that downregulation of ADCK1 expression inhibited tumor formation in nude mice and reduced the volume and weight of the tumors (Fig. 4B). In addition, we examined the tumors for ADCK1 expression by immunohistochemical staining. The results indicated that ADCK1 was downregulated in the in vivo tumors (Fig. 4C). In addition, we knocked down ADCK1 in SW620 cells and injected these cells into mice via the caudal vein to assess the formation of lung metastases. The experimental results demonstrated that downregulation of ADCK1 expression inhibited the formation of lung metastases in the mice (Fig. 4D, E).

### ADCK1 interacted with TCF4

**Fig. 5:**
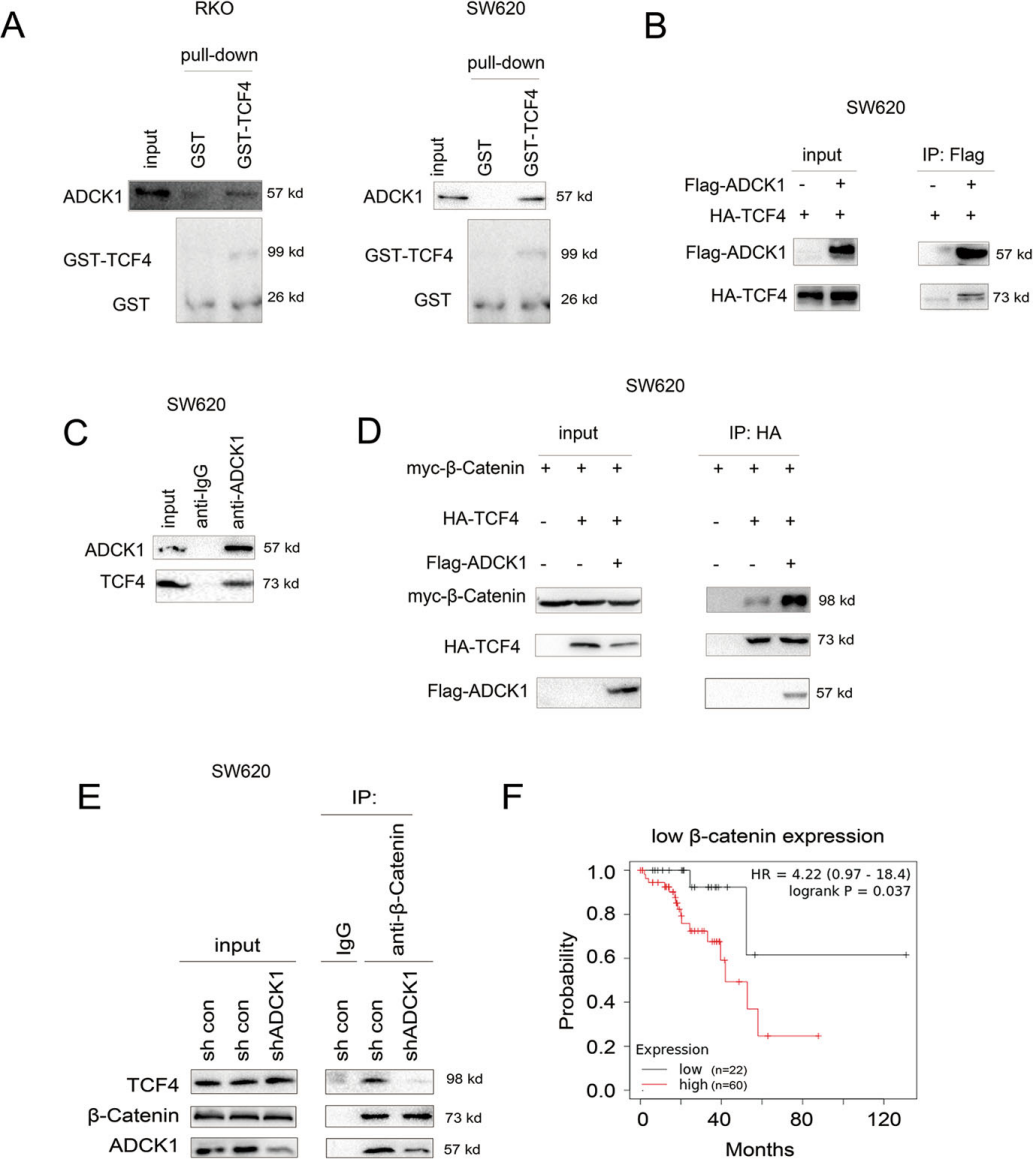
TCF4 interacted with ADCK1. **A** A GST pulldown assay was performed to examine the interaction between ADCK1 and the GST-TCF4 fusion protein in SW620 and RKO cells. **B** Co-IP was performed to examine the interaction between exogenously expressed ADCK1 and TCF4 in SW620 cells. **C** Co-IP was performed to examine the interaction between endogenously expressed ADCK1 and TCF4 in SW620 cells. **D** Co-IP was performed to examine the effects of ADCK1 overexpression on the interaction between β-catenin and TCF4 in SW620 cells. **E** Co-IP was performed to examine the effects of ADCK1 knockdown on the interaction between β-catenin and TCF4 in SW620 cells. **F** Survival analysis according to ADCK1 expression for the cohort with low β-catenin expression. The ADCK1 high expression group (high) had poorer survival than the ADCK1 low expression group (low).

As shown above, the size of the organoids decreased after downregulation of ADCK1 expression, and the size of enteral organoids was closely related to the activity of the Wnt/β-catenin signaling pathway^12^. Therefore, we inferred that ADCK1 controls the Wnt/β-catenin signaling pathway. Then, we studied the interaction of ADCK1 and the key components of the Wnt/β-catenin signaling pathway. It can be seen from the results of the GST pulldown experiment that ADCK1 interacted with the transcription factor TCF4 in the Wnt/β-catenin signaling pathway, and similar results were also found in SW620 and RKO cells (Fig. 5A). In the Co-IP experiment, we found that Flag-tagged ADCK1 (Flag-ADCK1) interacted with HA-tagged TCF4 (HA-TCF4) (Fig. 5B). In addition, the endogenous Co-IP experiment showed that endogenous ADCK1 and TCF4 formed a complex (Fig. 5C). Regarding the molecular mechanism, we found that ADCK1 promoted the interaction of β-catenin and TCF4 (Fig. 5D), while knockdown of ADCK1 inhibited the interaction of β-catenin and TCF4 (Fig. 5E). In addition, we examined whether the combination of ADCK1 and β-catenin expression predicts the survival of patients by mining the Kaplan–Meier Plotter database. In the cohort with lower β-catenin expression, higher ADCK1 expression indicated poorer survival (Fig. 5F).

### ADCK1 activated the Wnt/β-catenin signaling pathway to promote the malignant phenotypes of cancer cells

**Fig. 6:**
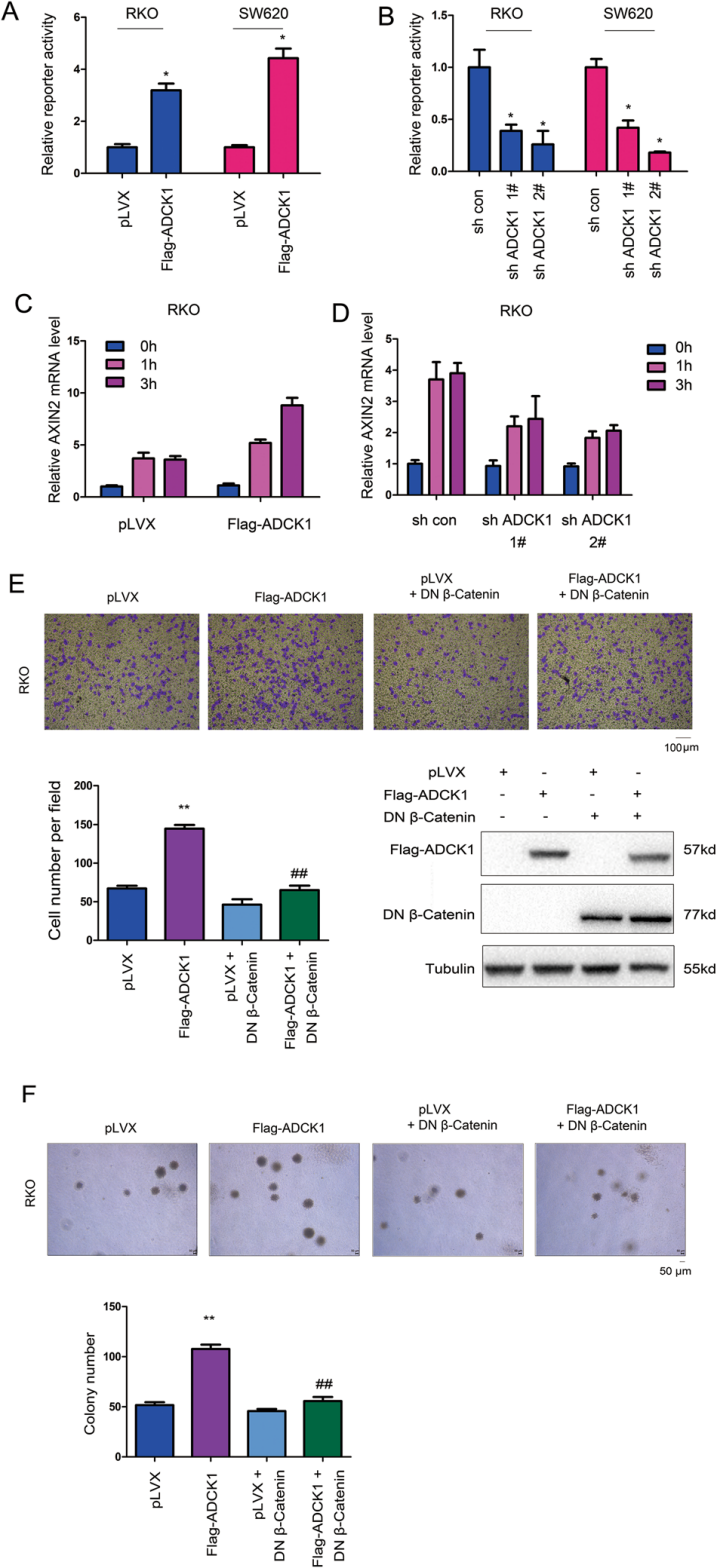
ADCK1 activated Wnt/β-catenin signaling. **A**, **B** The TOPFlash plasmid was used to examine the effects of ADCK1 on the transcriptional activity of the β-catenin/TCF4 complex. **C**, **D** qPCR was performed to examine the effects of ADCK1 on the expression of Axin2. **E** DN β-catenin abolished the effects of ADCK1 on the migration of RKO cells. The migrated cells were counted, and the expression of ADCK1 and DN β-catenin was confirmed using western blotting. **F** DN β-catenin abolished the effects of ADCK1 on the anchorage-independent growth of RKO cells. Fourteen days after seeding in the soft agar assay, the colony numbers were determined and analyzed. The scale bars were indicated. *P < 0.05; **P < 0.01; ^##^*P* < 0.01.

We explored the functions of ADCK1 in the Wnt/β-catenin signaling pathway. Overexpression of ADCK1 in colon cancer cells activated the Wnt/β-catenin signaling pathway reporter gene (Fig. 6A), and the activity of the reporter gene was downregulated after ADCK1 expression was inhibited (Fig. 6B). Moreover, we studied the impact of ADCK1 expression on the Axin2 expression level. The experimental results indicated that overexpression of ADCK1 cooperated with Wnt ligand stimulation to activate Axin2 expression (Fig. 6C). Downregulation of ADCK1 expression inhibited the induction of Axin2 expression (Fig. 6D). We also observed that the promotive effects of ADCK1 on cell invasion and anchorage-independent growth depended on the Wnt/β-catenin signaling pathway (Fig. 6E, F), indicating that ADCK1 promoted the malignant phenotypes of colon cancer cells by activating the Wnt/β-catenin signaling pathway.

## Discussion

The Wnt/β-catenin signaling pathway has a critical role in colon cancer and promotes the progression of epithelium from polyposis to adenoma^13–15^. Therefore, targeting this signaling pathway is of great importance in treating colon cancer. Considering the mutations in the critical upstream components of the Wnt/β-catenin signaling pathway in colon cancer, targeting the nuclear transcription complex β-catenin/TCF in this signaling pathway may be the ideal strategy. Several small-molecule inhibitors that target this transcription complex have shown therapeutic effects in cells and animal models^16^. In this study, we found upregulation of ADCK1 in colon cancer samples. ADCK1 promoted the in vivo tumor formation and metastasis of cancer cells. In the molecular mechanistic study, we found that ADCK1 promoted the activity of the transcription complex β-catenin/TCF. These studies indicated that ADCK1 might be an essential target for the treatment of colon cancer.

To study the expression pattern of ADCK1 in colon cancer, we collected colon cancer samples for immunohistochemical staining and scoring. We also assessed ADCK1 expression in early adenomas using a mouse adenoma model. The experimental results showed that upregulation of ADCK1 had already occurred in early adenomas. The Wnt/β-catenin signaling pathway is reported to be activated in early adenomas^17^. This observation showed that the expression of ADCK1 and the activation of the Wnt/β-catenin signaling pathway were synchronous and that ADCK1 was highly likely to be an indicator for the early diagnosis of intestinal cancer.

Another surprising finding of this study was that interfering with ADCK1 expression significantly inhibited the formation of organoids. The formation of organoids critically depends on the activated Wnt/β-catenin signaling pathway^18–20^. Downregulation of ADCK1 expression inhibited the formation of organoids, which revealed that downregulation of ADCK1 expression might negatively control the Wnt/β-catenin signaling pathway and the stemness of stem cells, as a result, inhibiting the activity of cancer stem cells.

This study also revealed the interaction of ADCK1 and TCF4. By interacting with TCF4, ADCK1 promoted the interaction of β-catenin and TCF4 and activated the expression of downstream genes. Interference with ADCK1 expression inhibited the transcriptional activity of the β-catenin/TCF4 complex and the expression of downstream genes. This finding indicated that interference with the interaction of ADCK1 and TCF4 with peptide fragments or small molecule inhibitors can suppress the activity of the transcription complex β-catenin/TCF4 and achieve a therapeutic effect.

In conclusion, this study identified the functions of ADCK1 in the progression of colon cancer and provided potential therapeutic targets.

## Experimental procedures

### Cell culture and transfection

SW620 and RKO cells were purchased from Shanghai Cell Bank of the Chinese Academy of Sciences (CAS). The cell culture medium was DMEM (Gibco) containing 10% serum (FBS, Gibco) and antibiotics. Cells were placed in a culture chamber that remained at 37 °C and was filled with 5% carbon dioxide. Cell transfection was conducted with Lipofectamine 2000 (Invitrogen) according to the instructions. Forty-eight hours later, the transfected cells were selected with 1 µg/mL puromycin (Sangon Biotechnology, A610593). After 7 days, viable cells were mixed, and the expression of ADCK1 was detected through western blot analysis. Cell lines were authenticated using STR profile analysis and used within 3–20 passages of thawing the original stocks.

### Clinical specimens

Clinical specimens were collected from Jingjiang People’s Hospital, and informed consent was received from patients. This study was approved by the ethics board of Jingjiang People’s Hospital. Specimens were embedded in paraffin after being fixed with formaldehyde and dehydrated with ethanol. Then, they were sectioned into 5 μm slices for subsequent use.

### Immunohistochemical scoring

The paraffin sections of four 8-week-old APC^min/+^ mice and human CRC tissue array (containing 74 normal tissues and 100 cancer tissues) were dewaxed with xylene and rehydrated through an ethanol gradient. Then, the sections were subjected to antigen retrieval with EDTA solution at 100 °C for 3 min in a pressure cooker, deactivation of endogenous peroxidase with 0.3% hydrogen peroxide, and blocking with 5% BSA. The paraffin sections were incubated with the ADCK1 antibody (HPA051012) overnight at 4 °C using an immunohistochemical kit (ZSGB-BIO, PV-8000), washed with PBST three times, and incubated with the secondary antibody at room temperature for two hours. Later, DAB (ZSGB-BIO, ZLI-9019) was used for color development for 2 min. After color development, the paraffin sections were stained with hematoxylin to identify cell nuclei. The paraffin sections were differentiated for 6 s with 1% hydrochloric acid/ethanol, and the liquid turned blue. The immunohistochemical score was evaluated with a Vectra 2.0 system to obtain the *H* value. The expression of the target gene in tumor tissues and normal tissues was evaluated based on the *H* values.

### Western blot

Cells were harvested with RIPA buffer. The protein concentration was determined using BCA. Then, SDS-PAGE was performed, and the proteins were transferred to a PVDF membrane. After blocking with 5% BSA at room temperature for 1 h, the membrane was incubated with the primary antibody for 4 h and sequentially with the secondary antibody for 1 h. Then, the signals were examined using an ECL kit. The information about the antibodies was: Anti-ADCK1 (Sigma, HPA051012), anti-GAPDH (Santa Cruz, sc-47724), ant-β-catenin (CST, 8480), anti-GST (Santa Cruz, sc-138), anti-Flag (Proteintech, 80010-1-RR), anti-c-Myc (Santa Cruz, sc-40), anti-Tubulin (Santa Cruz, sc-166729), anti-HA (Proteintech, 51064-2-AP), anti-TCF4(CST, 2569).

### Soft agar assay

The soft agar colony formation assay was conducted in a 12-well plate. The soft agar was divided into an upper layer and a lower layer. The agar in the lower layer was mainly used for coating the 12-well plate. The concentration of agar in this layer was 0.5%, and the serum concentration was 10%. The concentration of agar in the upper layer was 0.35% and the serum concentration was 10%; the agar in this layer was used to resuspend the cells. The agar was first heated to 37 °C, and the plate was then covered with the lower layer of agar. After this layer solidified, 2000 cells were added to the upper layer of agar, which was mixed and plated on top of the lower layer of agar. Cells were cultured for 14 days at 37 °C, photographs were taken, and a statistical conclusion was made.

### EdU assay

Cells were plated into a 96-well plate with 20000 cells in each well. The proliferation of the cells was detected using a Cell-Light EdU Apollo567 In Vitro Kit (RiboBio, C10310-1). A fluorescence microscope was used to acquire images for analysis. The percentage of positively stained cells was calculated.

### Invasion assay

Transwell inserts (Merck, CLS3398) were coated with Matrigel (BD Bioscience, 35623) containing no growth factors. DMEM containing 0.1% serum and 1000 cells was added to the upper compartment of the Transwell insert. DMEM that contained 10% FBS was added to the lower compartment. Forty-eight hours later, cells that had not infiltrated the membrane were removed with swabs. The infiltrated cells were stained with hematoxylin. Finally, the cells were photographed, and a statistical conclusion was made.

### Knockdown of ADCK1

Lentiviruses for interference with ADCK1 expression were purchased from Shanghai GeneChem Co., Ltd. Lentiviruses were incubated with cultured cells for 8 h and were then removed. After 24 h, puromycin (1 µg/mL) was added for screening. Viable cells were collected after 1 day to verify the expression of ADCK1. The shRNA sequences were as follows: sh ADCK1 1#, 5′-aaggcagtgctgcatgatggg-3′; sh ADCK1 2#, 5′-aaaggcggagattgtcctgtt-3′.

### Mouse model

C57BL/6J-*Apc*^Min^/J mice, which harbor a germline mutation in the tumor suppressor gene *Apc* and spontaneously develop intestinal polyps, were purchased from the Model Animal Research Center of Nanjing University and maintained in the animal facility at Nanjing Medical University. Genotyping was conducted by a routine allele-specific PCR assay. The C57BL/6J-*Apc*^*Min*^/J strain is highly susceptible to spontaneous intestinal adenoma formation. Homozygous mice are not viable. The C57BL/6J-*Apc*^*min*^ heterozygous mice develop adenomas throughout the intestinal tract at the age of 8–12 weeks.

### Organoids

Eight-week-old male C57BL/6J-*Apc*^Min^/J mice were used for crypt preparation. Isolation of crypts from tumor tissues was performed using 2 mM EDTA (Sigma, E9884) solution as previously described^12^. Isolated crypts were counted and pelleted. A total of 500 crypts were mixed with 50 mL of Matrigel (BD Bioscience, 35623) and plated in 24-well plates (Merck, CLS3398). After polymerization of the Matrigel (BD Bioscience, 35623), 500 µL of crypt culture medium (Advanced DMEM/F12 (Invitrogen)) containing the growth factors EGF (R&D, 236-EG), R-spondin 1 (R&D, 4645-RS), and Noggin (R&D, 6057-NG) was added.

### Subcutaneous tumor formation in nude mice

Eight-week-old male nude mice (SLAC, Shanghai) were randomly divided into two groups, each of which had four mice. The mice in one group were injected with SW620 cells (1*10^6^ cells/site) containing sh control, and the mice in the other group were injected with SW620 cells (1*10^6^ cells/site) containing sh ADCK1. The mice were killed after 5 weeks, the tumor tissues were excised and weighed, and ADCK1 expression was evaluated via immunohistochemistry. This study was approved by the ethical committee of the Jingjiang People’s Hospital and complied with the ethical regulations of the ethical committee of the Jingjiang People’s Hospital.

### In vivo metastasis experiment

Eight-week-old male nude mice (SLAC, Shanghai) were randomly divided into two groups, each of which had four mice. The mice in one group were injected with SW620 cells (1*10^6^ cells/site) containing sh control, and the mice in the other group were injected with SW620 cells (1*10^6^ cells/site) containing sh ADCK1. The mice were killed after 8 weeks, and the lung tissues were excised to detect the number of metastases through HE staining. This study was approved by the ethical committee of the Jingjiang People’s Hospital and complied with the ethical regulations of the ethical committee of the Jingjiang People’s Hospital.

### GST pulldown assay

SW620 and RKO cells were collected and split. They were centrifuged at 4 °C for 20 min at 12,000 rpm to produce a supernatant. The supernatant was incubated overnight at 4 °C with 10 µg of GST or GST-TCF4, and Sepharose 4B GST gel beads (GE healthcare) were then added and incubated for 4 h. The gel beads were washed with PBST buffer solution three times for 5 min each. Then, 30 μL of loading buffer was added to the gel beads and boiled at 100 °C for 5 min to produce a supernatant, which was later analyzed by western blotting.

### Co-immunoprecipitation (Co-IP)

SW620 and RKO cells were collected and split. They were centrifuged at 4 °C for 20 min at 12,000 rpm to produce a supernatant. The supernatant was mixed with the primary antibody and incubated overnight at 4 °C, and then Protein A gel beads (Thermo, 101090) were then added and incubated for 4 h. The beads were washed with PBST buffer solution three times for 5 min each. Then, 30 μL of loading buffer was added to the gel beads and boiled at 100 °C for 5 min to produce a supernatant, which was later analyzed by western blotting.

### Reporter assay

RKO and SW620 cells were seeded in 24-well plates. After 18 h, the cell confluence had reached to 70%. Then, 0.05 μg of TKRenilla, 0.1 μg of TOPFlash plasmid, and 0.5 μg of sh con plasmid or sh ADCK1 plasmid were transfected into the cells in each well with Lipofectamine 2000 (Thermo, 11668019). After 24 h, the lysate was added, and the activity of the reporter gene was measured with a dual-luciferase reporter assay system (Promega).

### Statistical analysis

All experiments in this study were performed in triplicate and error bars represent the standard deviation (±SD) of triplicate samples. Statistical analysis was conducted using GraphPad Prism (version 7.0). Comparisons between groups were performed using two-tailed independent sample Student’s *t*-tests analysis. Data were expressed as mean ± S.D, *P* < 0.05 was considered a significant difference (*P < 0.05; **P < 0.01; ***P < 0.001).
